# *In silico* prioritisation of microRNA-associated common variants in multiple sclerosis

**DOI:** 10.1186/s40246-023-00478-4

**Published:** 2023-03-30

**Authors:** Ifeolutembi A. Fashina, Claire E. McCoy, Simon J. Furney

**Affiliations:** 1 School of Pharmacy and Biomolecular Sciences, Royal College of Surgeons in Ireland, Dublin, Ireland; 2 SFI Centre for Research Training in Genomics Data Sciences, University of Galway, H91 TK33 Galway, Ireland; 3 FutureNeuro SFI Research Centre, Royal College of Surgeons in Ireland, Dublin, Ireland; 4 Genomic Oncology Research Group, Department of Physiology & Medical Physics, Royal College of Surgeons in Ireland, Dublin, Ireland

**Keywords:** Non-coding RNA, microRNA, 3′UTR binding sites, GWAS, microRNA–target prediction, Seed, Common variants

## Abstract

**Background:**

Genome-wide association studies (GWAS) have highlighted over 200 autosomal variants associated with multiple sclerosis (MS). However, variants in non-coding regions such as those encoding microRNAs have not been explored thoroughly, despite strong evidence of microRNA dysregulation in MS patients and model organisms. This study explores the effect of microRNA-associated variants in MS, through the largest publicly available GWAS, which involved 47,429 MS cases and 68,374 controls.

**Methods:**

We identified SNPs within the coordinates of microRNAs, ± 5-kb microRNA flanking regions and predicted 3′UTR target-binding sites using miRBase v22, TargetScan 7.0 RNA22 v2.0 and dbSNP v151. We established the subset of microRNA-associated SNPs which were tested in the summary statistics of the largest MS GWAS by intersecting these datasets. Next, we prioritised those microRNA-associated SNPs which are among known MS susceptibility SNPs, are in strong linkage disequilibrium with the former or meet a microRNA-specific Bonferroni-corrected threshold. Finally, we predicted the effects of those prioritised SNPs on their microRNAs and 3′UTR target-binding sites using TargetScan v7.0, miRVaS and ADmiRE.

**Results:**

We have identified 30 candidate microRNA-associated variants which meet at least one of our prioritisation criteria. Among these, we highlighted one microRNA variant rs1414273 (*MIR548AC*) and four 3′UTR microRNA-binding site variants within *SLC2A4RG* (rs6742), *CD27* (rs1059501), *MMEL1* (rs881640) and *BCL2L13* (rs2587100). We determined changes to the predicted microRNA stability and binding site recognition of these microRNA and target sites.

**Conclusions:**

We have systematically examined the functional, structural and regulatory effects of candidate MS variants among microRNAs and 3′UTR targets. This analysis allowed us to identify candidate microRNA-associated MS SNPs and highlights the value of prioritising non-coding RNA variation in GWAS. These candidate SNPs could influence microRNA regulation in MS patients. Our study is the first thorough investigation of both microRNA and 3′UTR target-binding site variation in multiple sclerosis using GWAS summary statistics.

**Supplementary Information:**

The online version contains supplementary material available at 10.1186/s40246-023-00478-4.

## Background

Multiple sclerosis (MS) is a complex chronic neuro-inflammatory condition that affects the central nervous system (CNS). This condition leads to periods of neurological disability in patients and is the cause of most non-traumatic neurological injury in young adults [1, 2]. MS pathogenicity is linked to environmental and genetic factors [3, 4]. Understanding the genetic contributions to MS could aid in identifying candidate biomarkers and in predicting disease aetiology and progression.

Genetic studies have uncovered part of the complexity of MS. More than 13 GWAS have been carried out on MS patients and control populations since 2007 [5]. Compared to earlier study designs, GWAS have contributed the most information about MS heritability, with over 200 non-MHC variants associated with the condition since the most recent GWAS [6]. Altogether, approximately 48% of MS heritability has been accounted for [6]. However, there are challenges in interpreting the causal variants within risk loci [5, 7]. In particular, non-coding variants are not often prioritised in these interpretation strategies.

**Fig. 1:**
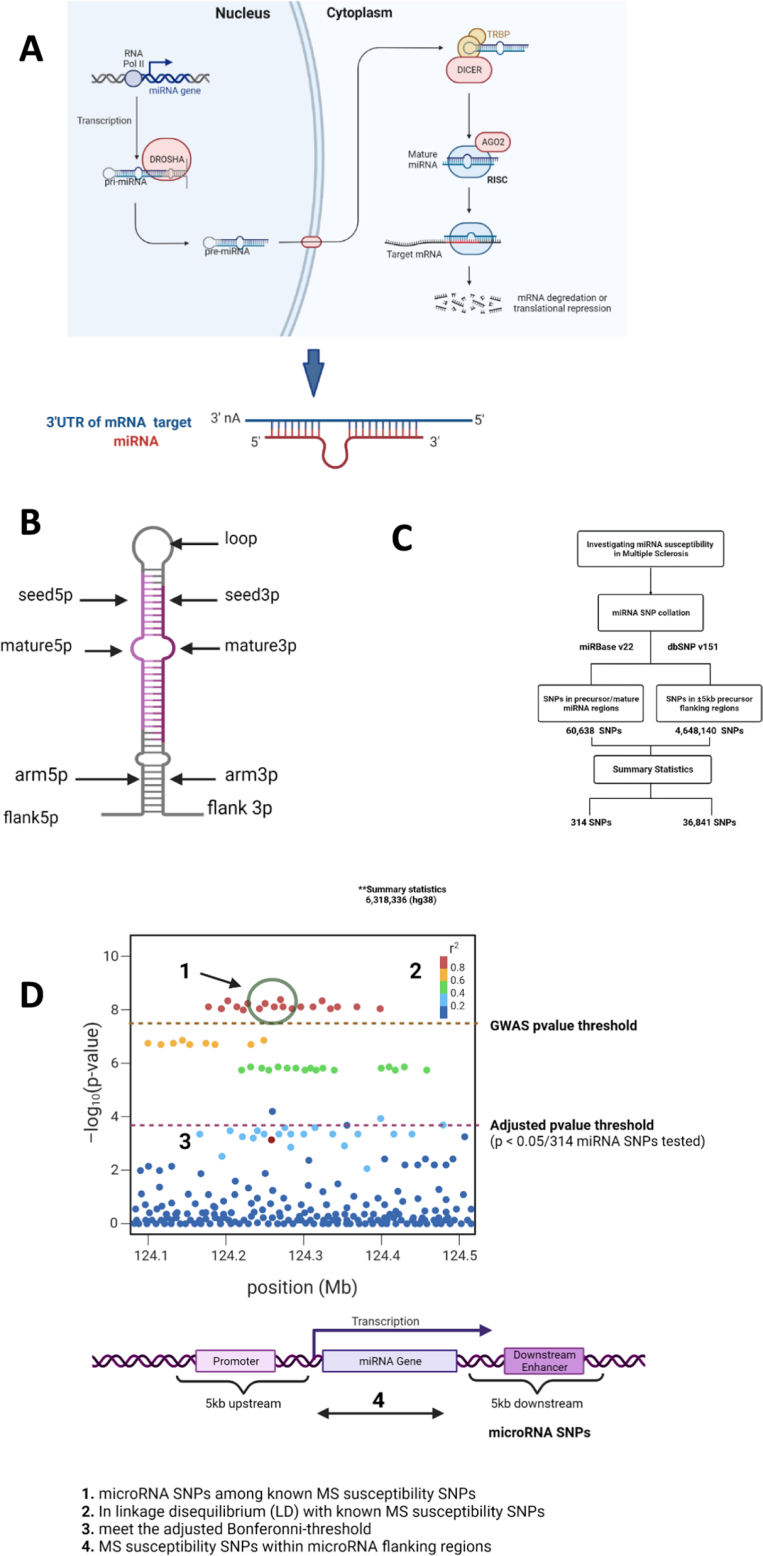
**A** Schematic representation of microRNA transcription and microRNA–mRNA interaction. microRNAs are transcribed from DNA sequences and processed by DROSHA from the primary structure to precursor structure and by Dicer into the mature sequence. These processed mature microRNA sequences then interact with mRNA targets, leading to mRNA degradation or translational repression. **B** microRNA precursor secondary structure. Altogether, we identified SNPs which are located within precursor, mature and 5-kb microRNA flanking regions. **C** Flowchart summarising our microRNA exploration procedure. We used summary statistics from the largest MS GWAS meta-analysis [6] and two publically available datasets to investigate microRNA-associated variation in MS. To capture variation within human microRNAs, we extracted the genomic coordinates of human microRNA precursor and mature regions from miRBase v22 and intersected these with all human variants recorded in dbSNP v151. In addition, we extended the precursor regions by 5 kb up- and downstream to incorporate SNPs within regulatory features (**D**). Overall, among the SNPs tested in the IMSGC’s meta-analysis, we identified 314 SNPs within microRNA precursor/mature regions and 36,841 SNPs in 5-kb flanking regions. **D** In our prioritisation process, we identified microRNA SNPs (1) among known MS susceptibility SNPs, (2) in strong Linkage Disequilibrium (LD) with known MS risk SNPs and (3) which meet the adjusted *p* value threshold for the 314 microRNA SNPs tested in the IMSGC meta-analysis. **A** and **D** adapted from “microRNA in Cancer” and “The Principle of a Genome-wide Association Study (GWAS)” in Biorender.com (2022). Retrieved from https://app.biorender.com/biorender-templates

MicroRNAs (miRNAs) are small ~ 22nt non-coding RNAs which are well conserved across organisms. They play central roles in post-transcriptional modification by binding the 3′UTR of their targets [8] (Fig. 1A), although other interactions have been reported within the 5′UTR, coding sequences and promoter sequences of target genes [9]. Interestingly, miRNAs have been shown to be dysregulated in immune cell subsets, cerebrospinal fluid (CSF) and plasma of MS patients, as well as in the MS mouse model, experimental autoimmune encephalomyelitis (EAE) [10, 11].

Despite strong evidence of microRNA dysregulation in MS patient samples and model organisms, there is limited literature on the role of microRNA variation in MS [12–15]. In contrast, variants in microRNAs and their processing machinery have been implicated in complex conditions such as cardio-metabolic conditions, colorectal cancer, glaucoma, Alzheimer’s disease and Parkinson’s disease [16–22].

We hypothesised that miRNA-associated variants are implicated in MS pathology. To explore this, we generated a bioinformatics pipeline to identify candidate MS susceptibility variants in microRNA genes and in the 3′UTR binding sites of microRNA targets using summary statistics from the most recent MS GWAS meta-analysis [6]. We then characterised and evaluated the effects of these variants using in silico methods and publically available datasets.

Therefore, our main objectives were collation, identification and characterisation of novel (a) microRNA gene susceptibility SNPs in MS and (b) microRNA 3′UTR binding site SNPs in MS.

## Results

### microRNA susceptibility SNPs

In order to investigate microRNA susceptibility SNPs in MS, we developed a prioritisation protocol as highlighted in Fig. 1. This protocol integrates common variation from dbSNP v151 with microRNA annotations from miRBase v22 [23, 24], in order to capture variation within precursor and mature microRNAs (Fig. 1B). We identified 60,638 SNPs in precursor/mature miRNA regions. These were obtained by intersecting 4573 mature and hairpin structures from miRBase with over 500 million SNPs from dbSNP v151 (Fig. 1C). This independent collation exceeds 56,911 microRNA SNPs (miR-SNPs) obtained from two older databases of microRNA variation, miRNASNPv3 and PolymiRTS [25, 26], and highlights the need to collate microRNA SNPs using more recent data.

Overall, we examined miR-SNPs which (a) are among known MS susceptibility SNPs, (b) are in linkage disequilibrium (LD) with known MS susceptibility SNPs, (c) meet a microRNA-specific adjusted Bonferroni threshold and finally (d) MS susceptibility SNPs which lie within microRNA flanking regions (Fig. 1D).

### MicroRNA variants among known MS SNPs

**Fig. 2:**
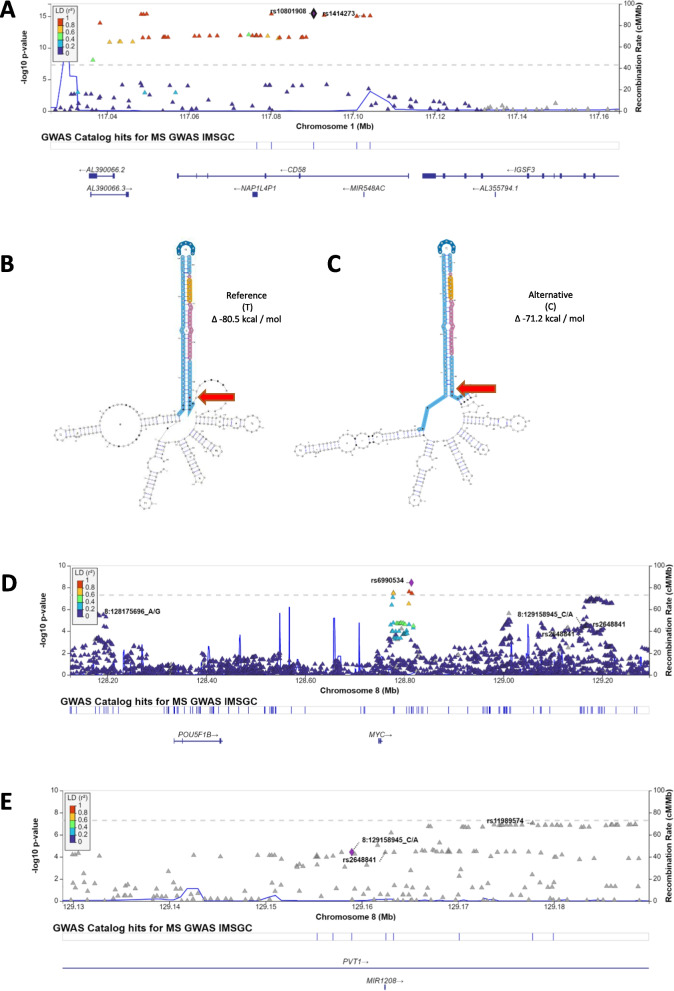
**A** Regional LocusZoom plot [62] showing high Linkage Disequilibrium (*r*^2^ > 0.9) between known MS susceptibility SNP rs10801908 (*CD58)* and our candidate SNP rs1414273, which lies in *MIR548AC.* Next, we highlight the predicted RNA secondary structure of the **B** reference sequence of hsa-mir-548ac compared to the **C** alternative (risk) allele. This figure shows the MEA prediction which has the greatest net change in free energy among the 3 models predicted by miRVaS (Additional file 1: Table S2). The arrow highlights the SNP rs1414273 (in red) located in the 3′ end (arm) of precursor sequence. Lower free energy measures indicate greater RNA stability; therefore, microRNA with the alternative risk allele is more thermodynamically stable than the reference allele. Candidate SNP rs2648841 is within genomic coordinates of *MIR1208*. **D** This variant represents a different signal from IMSGC SNPs rs6990534 and rs735542 (chr8:128175696) and **E** is not in LD with rs11989574, the peak SNP in its genomic region or rs1861842 (not shown) and rs759648 (chr8:129158945) which were implicated in African Americans and Europeans, respectively [53]. Although this SNP is below genome-wide significance (*p* = 3.86 × 10^–5^), its association with MS cannot be ruled out

Initially, we identified 314 SNPs within precursor/mature microRNAs that were tested within the 2019 GWAS summary statistics [6] (Additional file 1: Table S1). However, none of these microRNA variants were among the reported MS susceptibility SNPs. To investigate this further, we mapped miR-SNPs which were in linkage disequilibrium (LD) with the susceptibility SNPs, in order to capture tagging miR-SNPs within the MS susceptibility loci. 3 of the 314 miR-SNPs (rs1414273, rs7247237 and rs7247767) were in LD with the susceptibility SNPs (see Methods). Among these 3 miR-SNPs, rs1414273 is in high LD (*r*^2^ = 0.97) with known MS susceptibility SNP rs10801908 (*CD58*), meets the genome-wide *p* value threshold (*p* = 8.48 × 10^–16^) and lies within the 3′ end of precursor hsa-mir-548ac (Fig. 2A). Only recently has attention been drawn to rs1414273/*MIR548AC* in MS. Hecker et al. [14] found that rs1414273 (*MIR548AC)* decouples transcription of *CD58* and *MIR548AC*. Their findings support our independent prioritisation process for non-coding candidate variants which are in high LD with coding MS susceptibility SNPs.

Next, we examined the effect of rs1414273 on the structural conformation of hsa-mir-548ac using miRVaS [27]. This SNP significantly impacts the flank and arm of the microRNA in both the 5′ and 3′ directions. The fold energy changes across the centroid (CEN), minimum fold energy (MFE) and maximum estimate accuracy (MEA) models are presented in Additional file 1: Table S2. We have represented the MEA, which has a net change of − 4.4∆ in Fig. 2B and C.

In Fig. 2C, the risk allele (C) is predicted to create a more thermodynamically stable RNA secondary structure compared to the T allele. This could allow for more microRNA regulatory activity. In conclusion, rs1414273 is potentially associated with MS susceptibility and changes to MIR548AC stability.

### Genome-wide microRNA variants

Next, to specifically focus on miRNA-related variants, we carried out Bonferroni correction on the *p* value threshold, adjusting for the 314 miRNA SNPs that were tested in the summary statistics. This process yielded 6 candidate MS-associated miR-SNPs within the precursor, loop and seed regions of 4 microRNAs: *MIR548AC, MIR1208, MIR3135b* and *MIR6891* (Table 1). We established the functional implication of rs1414273 in *MIR548AC* in the previous step; therefore, we focused on the other 5 SNPs here. We examined the genomic context of the miR-SNPs and compared our candidates to reported GWAS signals.rs2648841 in the 3′ end of precursor MIR1208 (Table 1), which represents a signal separate from the IMSGC GWAS signals rs735542 (hg37 chr8:128175696), rs6990534 (*PVT)* (Fig. 2D)*,* the proximal peaks rs11989574 and rs759648 (chr8:129158945 in another European GWAS) (Fig. 2E). Similar to rs1414273 (*MIR548AC)* above, rs2648841 was not among the prioritised effects and therefore was not among suggestive, non-replicated or no data effects outlined by the IMSGC. However, we dropped this SNP due to its nominal *p* value compared to the lead SNPs and because no structural changes were predicted in 2 of 3 thermodynamic models (Additional file 2: Fig. S1).

**Table 1:** Annotation of the 6 miRNA SNPs that passed the Bonferroni-adjusted *p* value threshold (*p* < 0.05/314)

CHR: POS (hg38)	RSID	A1/A2	EUR_AF	Discovery GWAS * p* value	OR	miRNA	ADmiRE annotations	OR interpretation	Prioritisation results
1:116560027	rs1414273	C/T	0.1402	8.48E−16	1.2333	mir-548ac	Precursor_3PrimeEnd	risk	High LD, structural change
8:128150187	rs2648841	G/A,T	0.0179	3.86E−05	1.1322	mir-1208	Precursor_3PrimeEnd	risk	No structural change
6:31355288	rs17881225	G/C	0.0984	7.72E−05	1.2163	mir-6891	Precursor_Loop	risk	Signal in high LD *HLA* region
6:31355243	rs2276448	T/C	0.2366	7.73E−12	1.2517	mir-6891	3p_Seed	risk	Signal in high LD *HLA* region
6:31355235	rs2854001	G/A	0.2117	5.44E−38	1.5888	mir-6891	3p_Mature	risk	Signal in high LD *HLA* region
6:32749925	rs4285314	G/A	0.5318	7.1E−122	1.5134	mir-3135b	Precursor_3PrimeEnd	risk	Signal in high LD *HLA* region

ADmiRE [28] was used to identify the locations of these SNPs. In order of consequence, SNPs in the seed region > mature > loop > precursor ends. The association with MIR548AC was explored in the previous section. The structural consequence of MIR1208 SNP was explored, while the microRNA-binding ability of MIR6891 was examined in the context of the seed SNP. Discovery GWAS *p* values and ORs of these SNPs are also presented in context

Also among the 6 miR-SNPs (Table 1), 3 are within hsa-miR-6891-3p, a product of *MIR6891* (rs17881225, rs2276448 and rs2854001)*.* This microRNA is encoded within intron 4 of *HLA-B* and is co-transcribed with the mRNA*,* which is itself associated with MS susceptibility. Although these 3 candidate SNPs could have effects on target regulation, the GWAS signal is likely coming from the HLA variants identified by the IMSGC (rs2308655, rs3819284, rs1050556, HLA-B*52.01, HLA-B*38:01 and HLA-B*35:03). Among these 3 *MIR6891* SNPs, rs2276448 lies within the seed region of the microRNA and possibly has the most significant effect on its target regulatory function compared to the SNPs in the mature and precursor loop regions. We explored the target-binding consequences of the seed SNP rs2276448 (MIR6891) in Additional file text (Additional file 2: Fig. S2).

Finally, rs4285314 lies in the precursor 3′ end of *MIR3135b*, but is within the same susceptibility locus as the *HLA-B* variants, presenting the same challenge as the *MIR6891* variants.

Overall, having investigated the SNP in *MIR548AC*, we did not further prioritise any of the 5 Bonferroni-adjusted microRNA SNPs due to a) lack of predicted structural changes or b) high linkage disequilibrium in the MHC locus.

### microRNA flanking SNPs among known MS SNPs

Having explored our microRNA variants with regard to susceptibility SNPs, LD and adjusted *p* values, we expanded our definition of microRNA variants to include those within ± 5-kb flanking regions of the precursors, in a similar approach to that employed by Fang and colleagues [21]. By extending the miRNA precursor coordinates by ± 5 kb, we aimed to incorporate microRNA regulatory features that might be influencing their expression. We found over 4 million SNPs in ± 5-kb precursor flanking regions of miRNAs (Fig. 1C). In total, 36,841 of these were tested in the summary statistics (Fig. 1C). Among these, two variants proximal to hsa-mir-10399 and hsa-mir-4492 were among known susceptibility SNPs. rs10271373 and rs149114341 (chr11:118783424 hg37) are downstream of hsa-mir-10399 and hsa-mir-4492, respectively (Additional file 1: Table S5), and were annotated as intergenic SNPs in the 2019 GWAS meta-analysis. An enhancer sequence is reported around the coordinates of rs149114341. However, we were unable to characterise its effects on *MIR4492* using summary statistics only*.* rs10271373 maps downstream of *MIR10399* as well as to the 3′UTR binding site of *ZC3HAV1,* a gene that has been implicated in MS [6]. Therefore, this SNP was prioritised in our 3′UTR binding site analysis instead.

Altogether, our prioritisation process highlights rs1414273 (*MIR548AC*) as a candidate MS SNP among the other candidates (Additional file 1: Table S10).

### 3′UTR microRNA-binding site susceptibility SNPs

**Fig. 3:**
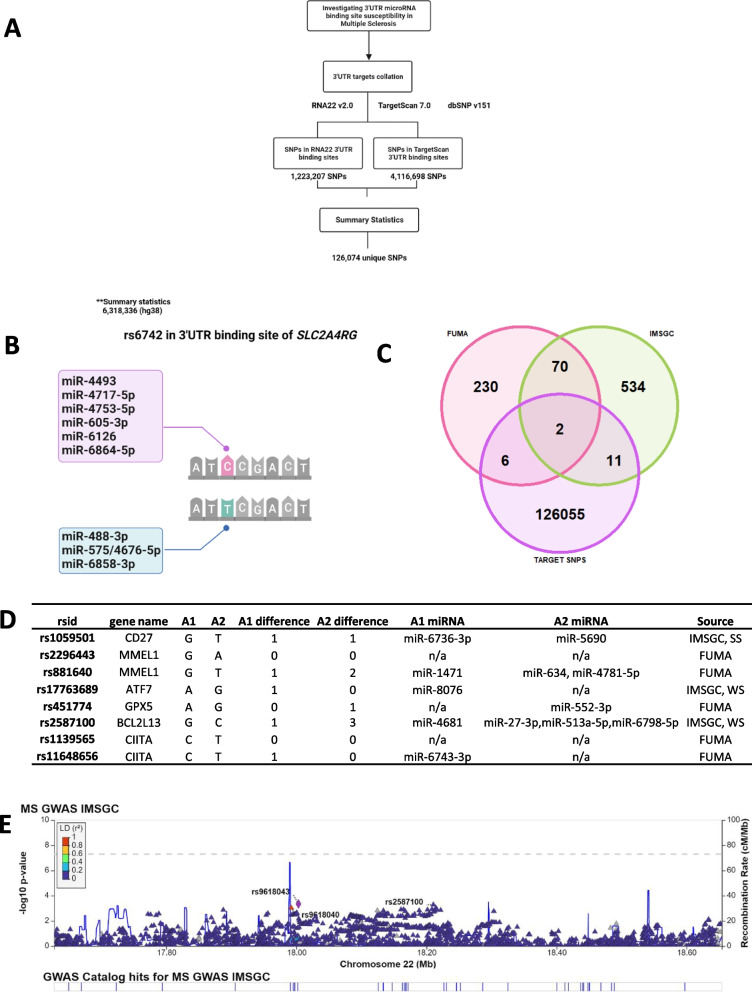
**A** Flowchart showing our 3′ UTR microRNA-binding site exploration pipeline using 3 publically available datasets and summary statistics provided by IMSGC (2019). 3′UTR variant collation was performed separately from microRNA variant collation. We obtained variants from dbSNP v151 and integrated these into the 3′UTR binding sites predicted by RNA22 v2.0 and TargetScan v7.0 (see Methods) (**B**)**.** Schematic showing the predicted effects of rs6742 on microRNA-binding ability to 3′UTR in SLC2A4RG. The C allele is expected to bind to 6 microRNAs differently to the T allele. Overall, we expect that the C allele is under stronger regulation than the T allele. **C** Venn Diagram showing the overlap between GWAS independent SNPs and our collection of 3′UTR SNPs which are in 3′UTR binding sites. Independent SNPs were identified through FUMA and a list of suggestive effects provided by the IMSGC. **D** Among the 19 independent SNPs from **C,** we tested the microRNA-binding ability of the 3′UTR binding sites containing 8 SNPs. Among these, 6 SNPs were found to cause microRNA-binding site changes in their 3′UTR sites. Here, we show the number of microRNAs that bind to the alternative and reference versions of the 3′UTR sequences, as well as the microRNAs that bind differently. The source column highlights which GWAS independent list the SNP has been output from. **E** LocusZoom regional plot showing the 3′UTR SNP rs2587100, which is independent, weakly suggestive, causes changes in microRNA-binding ability of BCL2L13 and is an eQTL for BCL2L13 in general monocytes and MS patient monocytes. This is our only candidate SNP which has MS patient specific eQTL evidence. No other SNPs in this region were prioritised among the genome-wide IMSGC SNPs. The highlighted intronic SNP rs9618043 (*CECR2)* is among the non-replicated SNPs (NR) from the IMSGC’s prioritised effects within this region (IMSGC Additional file 1: Table S6), while rs9618040 is not among the prioritised effects (intron *CECR2)*

Variants in the 3′UTRs of mRNAs could disrupt or create microRNA-binding sites, contributing to transcriptomic dysregulation. We explored variation in 3′UTR microRNA-binding sites that could be relevant to MS, by implementing a procedure similar to our microRNA variant collation (Fig. 3A).

3′UTR microRNA-binding sites were extracted from two microRNA–target prediction tools which implement different algorithms. Predictions from both TargetScan v7.0 and RNA22 v2.0 were used to capture microRNA–target interactions within the 3′UTRs [29, 30]. Among over 14 million RNA22-predicted binding sites, 1,223,207 sites were retained as they had the most significant *p* values per miRNA–target pair. In addition, over 15 million TargetScan-predicted binding sites were identified. Together, we found 1,223,207 RNA22 SNPs and 4,116,698 TargetScan SNPs after intersecting dbSNP v151 with the genomic coordinates of these 3′UTR binding sites (Fig. 3A). We collated 126,074 SNPs among the predicted 3′UTR binding sites which were also tested in the summary statistics.

### 3′UTR variants among known MS SNPs

Overall, we identified two [2] IMSGC susceptibility SNPs among our collated 3′UTR binding site variants. rs10271373 (*p* = 3.11 × 10^–9^, GWAS joint OR = 0.946) and rs6742 (*p* = 4.11 × 10^–14^, GWAS joint OR = 1.149) lie within the predicted 3′UTR microRNA-binding sites of *ZC3HAV1* and *SLC2A4RG*, respectively. We used TargetScan v7.0 to analyse miRNA-binding changes in reference versus variant 3′UTR sequences for these 2 candidate mRNAs. Of the 2 susceptibility SNPs, we observed changes in miRNA-binding ability for rs6742 only. In short, rs6742 changes which miRNAs can bind to the *SLC2A4RG* 3′UTR of both alleles (Additional file 1: Table S6, Fig. 3B). Overall, the risk allele of rs6742 appears to be under tighter miRNA regulation than the T allele, with a net change of + 3 miRNA interactions (Fig. 3B).

### ***3***′***UTR variants among independent SNPs***

We postulated that more candidate 3′UTR binding site MS SNPs exist, but were not prioritised as susceptibility SNPs in the 2019 meta-analysis. Therefore, to broaden our 3′UTR binding site candidates, we first identified independent SNPs from the IMSGC’s Additional file [6] (Additional file 1: Table S11). In total, we extracted a list of 201 independent genome-wide SNPs and 416 independent weakly and strongly suggestive (299 WS and 117 SS) SNPs. For some of these suggestive SNPs, their joint *p* values were greater than their discovery *p* values; however, they did not meet genome-wide significance, while others replicated significantly in only one dataset [6] (Additional file 1: Table S11). We then explored whether any of our 126,074 3′UTR SNPs, which were tested in the summary statistics were among these independent SNPs from IMSGC. On intersecting both datasets, we identified 13 3′UTR SNPs within the IMSGC’s independent SNPs (Additional file 1: Table S7, Fig. 3C).

To expand the methodology used to identify independent SNPs within the summary statistics, we uploaded the IMSGC GWAS summary statistics into FUMA Webtools [31]. While the IMSGC applied stepwise conditional regression to their discovery and replication cohorts to identify independent effects, FUMA uses PLINK’s [32] clumping procedure to rank independent and lead SNPs from GWAS summary statistics. We identified 318 independent SNPs from the FUMA web tools. Eight of our 3′UTR SNPs were among the FUMA independent SNPs (Additional file 1: Table S7, Fig. 3C). Additionally, two [2] 3′UTR independent SNPs were shared by both FUMA and the IMSGC (Fig. 3C). Altogether, we identified 19 3′UTR SNPs among the independent SNPs (Additional file 1: Table S7).

We set out to investigate these 19 3′UTR SNPs through *in silico* methods and publically available functional evidence (see Methods). Other studies [33–35] have proposed criteria to validate microRNA–target interactions. These can be summarised as (1) demonstration of co-expression, (2) direct interaction between miRNA and region on target, (3) gain and loss experiments to show target protein interaction and (4) predicted changes have biological functions. We incorporated these approaches into our prioritisation process (see Methods). In short, functionally relevant 3′UTR SNPs are likely to change miRNA–target interactions at the 3′UTR binding site, act as eQTLs for the targets in MS relevant tissues (e.g. PBMCs, lymphocytes) and have the relevant microRNAs expressed in the same MS relevant tissues. We investigated these using the FiveX browser for eQTL catalogue, an MS eQTL dataset from the IMSGC, RegulomeDB v2.07 and FANTOM5 [6, 36–38] (Additional file 1: Table S11). These criteria are highlighted in Additional file 2: Fig. S3.

Among our 19 candidates, we excluded 11 for the following reasons. Two had been assessed in the susceptibility SNP process, 7 had been reannotated as intronic and the relevant 3′UTR sequences were unavailable for 2 SNPs. Therefore, we carried out microRNA gain/loss on 8 independent SNPs using TargetScan 7.0 (Additional file 1: Table S7). Among these 8, we found that 6 SNPs (rs1059501 *CD27*, rs881640 *MMEL1*, rs2587100 *BCL2L13*, rs11648656 *CIITA*, rs17763689 *ATF7* and rs451774 *GPX5*) change the microRNA-binding ability of 3′UTRs (Fig. 3D, Additional file 1: Table S8).

The 3′UTR of *BCL2L13* has the greatest changes in microRNA binding due to rs2587100 (GWAS joint OR = 3.43 × 10^–05^, joint *p* value = 1.049). The G allele (risk allele) binds to miR-4681 while the C allele binds to miR-27-3p, miR-513a-5p and miR-6798-5p, suggesting that the 3′UTR sequence which includes the risk allele is possibly under less regulation (Fig. 3D). rs2587100 was among the weakly suggestive effects (IMSGC), but has strong functional support for its potential role. rs2587100 is also the only SNP among our 6 candidates which is an eQTL for the target gene in non-MS and an MS patient dataset (see Methods). However, the effect of the eQTL is not consistent across the non-MS and 1 MS dataset. It decreases BCL2L13 expression in non-MS PBMCs and monocytes, and increases BCL2L13 expression in MS PBMCs and monocytes, and its relevant microRNAs are also expressed in monocytes [6, 38–40] (Additional file 1: Table S9, Additional file 1: Table S11). We have presented the genomic context of this SNP in Fig. 3E. Apart from rs9618043 *CECR2,* which is among the IMSGC’s non-replicated SNPs, no other SNPs in this region were among the prioritised genome-wide IMSGC SNPs.

**Fig. 4:**
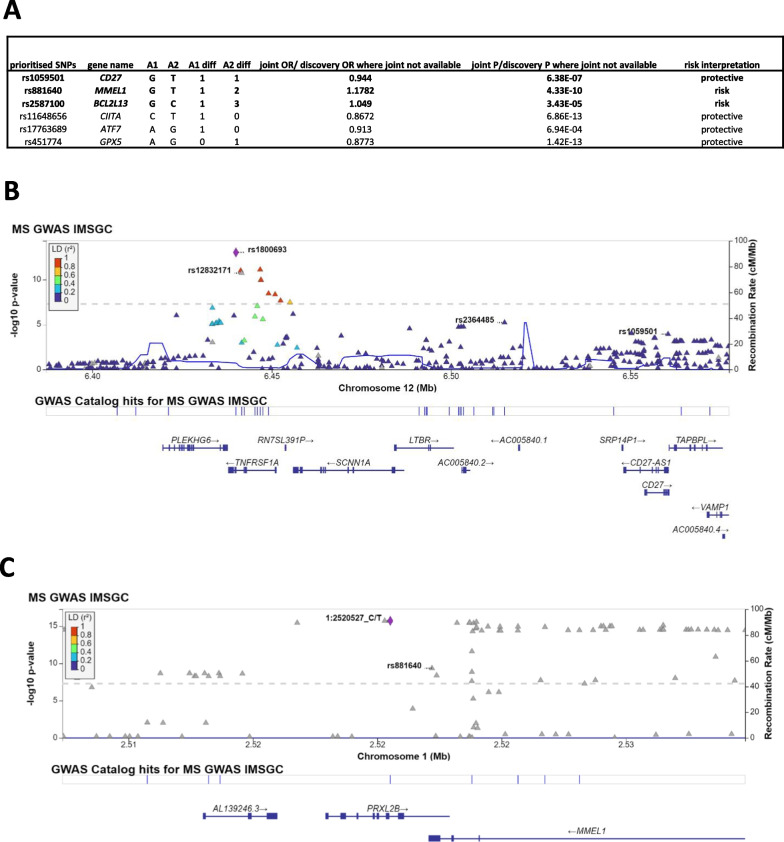
**A** Table showing the *p* values and odds ratio (OR) for the 6 independent 3′UTR independent SNPs. Among these, only 3 (highlighted in bold) meet 3 of the microRNA–target validation criteria (see Methods, Additional file 1: Table S9). Joint *p* values (from IMSGC’s discovery and replication processes) are available for the IMSGC independent (suggestive) SNPs, but not for those identified by FUMA, as these were not among the suggestive effects. For the latter group, we have showed the discovery *p* values and ORs. **B** LocusZoom plots showing regions around our other 2 functionally relevant SNPs (rs2587100 is in Fig. 3E). We have highlighted the 3′UTR SNPs in rs1059501 *(CD27) and* rs881640 (*MMEL1).* Our candidate SNP rs1059501 is independent from the IMSGC susceptibility/genome-wide SNPs (rs1800693, rs2364485, rs12832171) in that region and was ranked as strongly suggestive in the IMSGC stepwise regression. **C** Our candidate SNP rs881640 is independent from the IMSGC susceptibility/genome-wide SNP (chr1:2520527(hg37); rs6670198) in that region

Only two [2] other independent 3′UTR SNPs (rs1059501 and rs881640) met the functional validation criteria (Fig. 4A, Additional file 1: Table S9). The significant eQTL activity or microRNA expression for rs11648656, rs17763689 and rs451774 appears to not be relevant for MS tissues (Additional file 1: Table S9, Additional file 2: Fig. S4). Therefore, we prioritised rs1059501 and rs881640 and have shown their genomic context in Fig. 4B and C. rs1059501 (*CD27)* is independent from the IMSGC susceptibility SNPs (rs1800693, rs2364485 and rs12832171) in that region and was ranked as strongly suggestive in the IMSGC stepwise regression. Both the protective (*G*) and alternative allele (*T*) lose and gain one miRNA, respectively (Fig. 3D). The microRNAs gained/lost due to this SNP are expressed in monocytes and haematopoietic cells (Additional file 1: Table S9) [38], while the SNP has been shown to decrease CD27 expression in T-cells and LCLs, and increase CD27 in monocytes and brain tissue [41–45]. Finally, rs881640 is independent from the IMSGC genome-wide SNP [chr1:2520527(hg37); rs6670198] in that region. Its G (risk) allele binds to miR-1471, but not to miR-634 or miR-4781, which are recognised by the T allele. Therefore, we expect that 3′UTR sequence containing the G allele is likely under less regulation than the T allele. This SNP has been shown to decrease MMEL1 expression in blood, monocytes and T-cells [41, 46, 47].

Overall, we highlighted that the impact of increased regulation of *SLC2A4RG* due to the new miRNA interactions could be significant. In addition, 3 independent SNPs in 3′UTRs of *BCL2L13, CD27* and *MMEL1* meet multiple miRNA:target interaction criteria. Therefore, we have presented evidence that these SNPs could be involved in MS pathogenesis and should be prioritised for future investigation.

## Discussion

In this study, we presented evidence that microRNA-associated variants could be implicated in MS. Our analysis is the first systematic exploration of both microRNA and 3′UTR target-binding site variation in MS, using GWAS summary statistics. By using the most recent meta-analysis [6], we harnessed the largest MS GWAS resource available to test our hypothesis. Altogether, we identified 30 candidate microRNA-associated variants from our collation procedure. Those variants meet a microRNA-specific Bonferroni-corrected threshold, are in LD (Linkage Disequilibrium) with known susceptibility SNPs or are suggestive SNPs from the IMSGC GWAS [6], whose microRNA functions had not been evaluated previously. We prioritised 1 of 8 miR-SNPs, rs1414273 (*MIR548AC)*, and 4 of 22 SNPs in 3′UTR microRNA-binding sites of *SLC2A4RG* (rs6742)*, CD27* (rs1059501)*, MMEL1* (rs881640) and *BCL2L13* (rs2587100), based on structural and functional predictions. Therefore, these 5 SNPs are our top candidate microRNA-associated variants which could play a role in MS pathogenesis.

Our work successfully incorporates multiple microRNA prioritisation methods used elsewhere [12, 19, 21, 22]. The most relevant comparison to our results is a study which implicated 11 microRNAs in MS susceptibility [48]. We noted one major difference in methodology. Hecker and colleagues [48] analysed all SNPs within microRNA stem loop and Drosha cleavage sites which are close (< 250 kb) to the 233 IMSGC GWAS SNPs, irrespective of their presence in the summary statistics. Therefore, association analysis-based *p* values were not factored in within their prioritisation process. Among of their 12 candidate SNPs, 6 had been tested in the GWAS and were not Bonferroni-corrected. However, both studies identified hsa-mir-548ac and hsa-mir-4492 as candidate MS microRNAs. Another important comparison is against a microRNA GWAS study performed on a paediatric MS cohort [12]. Rhead et al. [12] adjusted the *p* value threshold for microRNA variants within a paediatric cohort of MS patients, but did not identify any significant SNPs. To follow up, those authors used MIGWAS [49] to identify enrichment of candidate microRNA–target network signals. Alternatively, we examined our candidates individually, to characterise effects of the variants on the functions of microRNAs and targets directly.

Parallel to other studies, we successfully examined the effect of risk alleles through *in silico* methods [22, 50–52]. Our secondary structure prediction spotlighted that the risk allele for rs1414273 is expected to yield higher MIR548AC levels. Interestingly, rs1414273 has been shown to decouple the transcription of miR-548ac from its host gene CD58, leading to increased levels of miR-548ac [14]. This is in line with our secondary stability model. Despite rs1414273 [chr1:117102649 (hg37), 0.14 EUR MAF] being significant in the discovery cohort of the IMSGC meta-analysis, it was not among the effects prioritised for replication. It also appears not to have been captured among the 46 SNPs within that haplotype between the two replication datasets [6] (Additional file 1: Table S11). Conversely, the IMSGC’s prioritised effect SNP rs10801908 alone might paint an incomplete picture, due to the presence of *MIR548AC* within the first intron of *CD58* and the strong linkage between rs10801908 and rs1414273. However, there is limited research into the role of *MIR548AC* in immunological conditions. Next, although our candidate rs2648841 did not change the structural conformation of miR-1208*,* another *MIR1208* SNP rs1861842 has been associated with MS in African Americans [53], implicating the microRNA further.

In additional *in silico* experiments, we identified changes to MIR6891’s binding ability, which could lead to functional changes to the mRNA. However, because *MIR6891* lies within an intron of *HLA-B,* a class I MHC molecule with protective MS SNPs [54, 55], it is challenging to segregate the MHC signal from the microRNA signal using only summary statistics. While *MIR6891* seed SNP rs2276448 itself has not been assessed in MS, miR-6891-3p is linked to changes in macrophage-driven inflammation [56]. Our microRNA gain/loss analysis also showed that the risk allele of rs6742 in *SLC2A4RG* is likely under stronger microRNA regulation than the other allele. This is supported by the IMSGC’s 2019 study, where SNPs within the rs6742 susceptibility locus were all associated with reduced SLC2A4RG expression in CD4 + T-cells in an MS cohort [6] (Additional file 1: Table S11). SLC2A4RG functions as a transcription factor for SLC2A/GLUT4, which is among the glucose carriers that are upregulated following lymphocyte activation [57]. This highlights a possible link between SLC2A4RG dysregulation in CD4 + T-cells and T-cell activation.

After broadening our search for independent SNPs through FUMA, we identified changes to the microRNA-binding ability of *CD27, MMEL1* and *BCL2L13* due to rs1059501, rs881640 and rs2587100, respectively. This highlights the value of using different methods to identify independent SNPs. The eQTL rs2587100 drives increased expression of *BCL2L13* in MS patients [6] (Additional file 1: Table S11) and aligns with our microRNA gain/loss experiment which shows that the risk allele is under less regulation than the C allele. *BCL2L13* has been linked to mitophagy [58]; therefore, investigation of this upregulation in monocytes could be important. However, surprisingly, the non-MS eQTLs for both *BCL2L13* and *MMEL1* reduce their expression [38–40]. Genotyping these SNPs directly in MS patients could clarify the true direction of this eQTL.

Next, at least one other group has incorporated flanking regions in microRNA-specific GWAS, in order to explore regulatory features which may influence microRNA transcription [21]. Our identification of a risk SNP in an enhancer-like domain [59] flanking *MIR4492* suggests the regulation of these microRNA genes by other factors. Expression of this microRNA within B-cells is proposed to be altered due to Epstein–Barr Virus (EBV) infection, which has been shown to increase MS risk significantly [4, 13]. The effect of this enhancer SNP on MIR4492 expression in MS patients should be investigated further, especially in the context of EBV infection.

The main challenges with interpreting our findings are the long-range LD in the MHC region, limited microRNA annotations and the ability of microRNAs to bind to multiple targets. We identified consequences of seed SNP rs2276448 (*MIR6891)*, but could not confirm its independence from HLA-B SNPs (rs2308655, rs3819284, rs1050556, HLA-B*52.01, HLA-B*38:01 and HLA-B*35:03) using only publically available data. Furthermore, we were unable to measure the effect of multiple candidate SNPs on microRNAs or their targets by using only summary statistics. We also could not annotate the flanking SNPs which exceeded the 2-kb region stipulated by miRVaS. This is a challenge with microRNA tools such as miRVaS, as promoter information is not often available for intergenic microRNAs [60]. This means that the microRNA mapping tools are not fully powered to identify SNPs in enhancer regions, transcription start sites, among others; therefore, this needs to be accounted for in downstream analysis. Finally, experimental validation of our predicted changes in *MIR6891, SLC2A4RG*, *CD27, MMEL1* and *BCL2L13* will be necessary in the future due to the limitations of microRNA–target prediction algorithms. Finally, this study was limited to publically available data; therefore, the eQTL data were sourced from multiple studies.

## Conclusions

Altogether, we identified 30 candidate microRNA-associated variants through systematic analysis of MS GWAS summary statistics. We prioritised 1 microRNA SNP and 4 3′UTR binding site SNPs based on the effects of the MS variants on their function, structure or regulatory abilities. Our *in silico* work helps to bridge the gap between MS GWAS and microRNAs implicated in MS.

## Methods

### Summary statistics

Summary statistics from the most recent GWAS meta-analysis [6] on MS patients were requested from the IMSGC through the webpage (https://imsgc.net/). In short, over 8 million SNPs were imputed and tested for 47,429 MS cases and 68,374 control subjects by the consortium.

Genomic coordinates for all summary statistics (including autosomal and non-autosomal SNPs) were provided in hg37. We lifted over to hg38 using Ensembl’s [61] Assembly Converter for downstream hg38 SNP integration. We visualised all regional associations in LocusZoom’s web platform [62]. An overview of the pipeline and tools is presented in Additional file 2: Fig. S5.

### Text mining

Prior to collating microRNA SNPs, we wanted to test whether the microRNA-associated variant databases PolymiRTS [26] and miRNASNP v3 [25] were up to date. miRNASNP v3 contains SNPs in microRNA seed and precursor regions, target 3′UTR SNPs as well as predictions of miRNA gain/loss based on these 3′UTR SNPs. PolymiRTS was last updated in 2014 and contains microRNA seed regions from miRBase v20 and 3′UTR sequences for CLASH validated targets. Altogether, this resulted in the collation of 56,911 SNPs. We compared microRNA SNPs from the literature to those within the databases. Specifically, the term “microRNA” was used in PubMed’s eFetch commandline tool, to obtain abstracts for all relevant papers published between 2014 and 2021. We then extracted rsids from these abstracts and manually confirmed whether the SNPs were referring to the microRNAs. Following this manual check, we tested the presence of those text mined SNPs within PolymiRTS and miRNASNP v3. The absence of recent miR-SNPs from the databases guided our independent collation step.

### Collation of microRNA–associated variants

Variants within microRNA precursor and mature regions as well as those in ± 5-kb flanking regions were collated. To achieve this, genomic coordinates of microRNA precursor and mature sequences were downloaded from miRBase v22 [63] (https://www.mirbase.org/ftp/CURRENT/genomes/hsa.gff3) and intersected with genomic coordinates from the full dbSNP v151 [23] catalogue using BEDTools [64].

Primary transcripts of intergenic microRNAs are not well characterised. However, several studies have shown that flanking regions between ~ 1 kb and 10 kb are likely to contain transcription start sites, CpG islands, expressed sequence tag (EST)- and transcription factor (TF)-binding sites [21, 60, 65]. By extending the microRNA precursor coordinates by ± 5 kb, we aimed to incorporate microRNA regulatory features that might be influencing microRNA expression. We extracted sequences marked as “microRNA_primary_transcript” from the miRBase v22 gff file. These represent precursor sequences. These coordinates of these transcripts were extended by 5 kb in both directions using the BEDTools suite.

### microRNA SNPs tested in summary statistics

We intersected the collated microRNA and ± 5-kb flanking SNPs with the lifted over summary statistics. Bonferroni correction was applied on microRNA SNPs found among the summary statistics. The *p* value thresholds were adjusted as follows: microRNA SNPs (0.05/314) and ± 5-kb flanking SNPs (0.05/36,841).

### microRNA-associated SNPs among susceptibility SNPs

In total, 200 non-MHC autosomal SNPs were significantly associated with MS in the most recent meta-analysis [6]. Those susceptibility SNPs can be obtained from Additional file tables (Additional file 1: Table S7) of that paper. We intersected the genomic coordinates of our collated microRNA, ± 5-kb flanking and 3′UTR target SNPs with these susceptibility SNPs. Nominally significant SNPs which did not meet the genome-wide threshold were extracted from the IMSGC [6] Additional files (Additional file 1: Table S14). These were merged with the susceptibility SNPs to create a dataset of independent SNPs. The file names for all datasets extracted from the IMSGC study are listed in Additional file 1: Table S11.

### microRNA-associated SNPs in LD with susceptibility SNPs

We aimed to capture entire susceptibility loci by mapping variants in linkage disequilibrium with the susceptibility SNPs. For this step, both the effect SNPs and discovery SNPs provided in Additional file 1: Table S7 of the IMSGC analysis [6] were used as susceptibility SNPs. We obtained all variants in LD with these susceptibility SNPs through Ensembl’s perl API, specifically using 1000 genomes EUR subset as the reference population. LD information was available for 174 of the 201 non-MHC susceptibility SNPs. This step was carried out for both sets of microRNA variants and the 3′UTR variants within the summary statistics.

### microRNA–target gain/loss analysis

TargetScan 7.0 prediction algorithm was used locally to analyse 3′UTR binding changes in variant vs reference microRNA seed sequence. The SNP position seed sequence was located within microRNA reference FASTA sequences using SeqKit [66], which we also used to swap the reference and alternative alleles. These seed sequences were then replaced within the TargetScan miR_Family_Info.txt, while the 3′UTR file was retained. The transcripts were mapped to gene names, and differences between the predictions for both microRNA sequences were analysed in R.

### microRNA variant effect prediction

ADmiRE and miRVaS were used to predict the location and effects of microRNA variants, respectively.

Oak et al. [28] provide microRNA annotation tab files in the ADmiRE repository. These were formatted into BED files and lifted over to hg38. The BED files were intersected with vcf files of microRNA variants of interest. This procedure was implemented by Tyc and colleagues [67]. miRVaS [27] runs predictions within 2000 nucleotides of microRNA coordinates using underlying tools VARNA and RNAfold [68, 69]. miRVaS is available online, or in local Windows or Linux packages. SNP coordinates were input into miRVaS using the required format, and predictions were run based on the hg38 reference file and miRBase v21.

### Collation of 3′UTR target-binding variants

#### TargetScan variants

We intersected TargetScan v7.0 [30] bedfiles containing genomic coordinates of all predicted sites with the UTR genome coordinates available on TargetScan. Coordinates in the former set of files were lifted over from hg19 to hg38 prior to this intersection. This intersection resulted in a collection of TargetScan-predicted binding sites within 3′UTRs. The binding site coordinates in the resulting bedfile were intersected with dbSNP 151 variants (Fig. 3A) for a final dataset of 3′UTR SNPs within binding sites predicted by TargetScan v7.0. All our intersection steps were carried out using combinations of VCFtools, BEDtools and SAMtools [70–72].

#### RNA22 variants

There were over 83 million predicted binding sites available from RNA22 [29] v2.0. We chose the minimum prediction *p* values for each microRNA–target pair predicted to interact at 3′UTRs, leading to ~ 14 million pairs (*p* value < 0.0314). Next, a custom R script was used to convert the cDNA coordinates to genomic coordinates. These were intersected with dbSNP v151 to get 1,223,207 SNPs (Fig. 3A). Additional file 2: Fig. S6 shows the overlap between targets predicted by TargetScan and this RNA “best probability” subset.

The dataset containing the union of binding site SNPs from TargetScan and RNA22 was used to test the presence of 3′UTR SNPs among the summary statistics.

### 3′UTR susceptibility SNPs

After intersecting the coordinates of the collated SNPs within 3′UTR binding sites with those of the susceptibility SNPs from the IMSGC, we identified 5 3′UTR binding sites among them. Three of the transcripts relevant to the predicted microRNA-binding sites had been archived by Ensembl. Therefore, those SNPs could no longer be annotated on those transcripts. Joint *p* values and ORs for the two [2] candidate SNPs were obtained from Additional files (Additional file 1: Table S7) of the IMSGC 2019 meta-analysis.

### Identification of independent 3′UTR SNPs

The IMSGC identified SNPs among their prioritised effects which were independent of the lead SNPs in those regions, but did not reach genome-wide significance, and were not replicated or whose joint *p* values were greater than the discovery *p* values. These SNPs are in Additional file 1: Tables S6 and S14 of the IMSGC’s paper (Additional file 1: Table S11).

They identified 201 genome-wide (GW) independent effect SNPs, 117 strongly suggestive effect SNPs and 299 weakly suggestive effects. We collated a list of the weakly and strongly suggestive SNPs from these tables.

To identify independent SNPs separately from IMSGC’s process, summary statistics were input into FUMA’s [31] online platform (https://fuma.ctglab.nl/). FUMA [31] uses PLINK’s [73] clumping procedures to highlight independent SNPs and lead SNPs. The intersection between both sets of independent SNPs was used for the functional prioritisation pipeline.

Among the 19 independent SNPs identified, the transcripts proposed to contain 7 3′UTR SNPs had been archived, and those SNPs had been reclassified as intronic SNPs, and the relevant 3′UTR sequences were unavailable for 2 (Additional file 1: Table S7). In addition, 2 independent SNPs had been assessed in the susceptibility SNP analysis, leaving 11 for microRNA gain/loss analysis in the next step.

### microRNA–target functional pipeline

A number of groups [33–35] have proposed criteria to validate microRNA–target interactions. We have summarised these as (1) demonstration of co-expression, (2) direct interaction between miRNA and region on target, (3) gain and loss experiments to show target protein interaction and (4) predicted changes have biological functions. We have adapted these to suit our bioinformatics approach. By using in silico microRNA gain/loss, we will assess the direct interaction condition (condition 2). We will also use publically available eQTL data to meet condition 4 (changed biological functions) and are using microRNA expression data in combination with the eQTL data to test condition 1. In short, relevant 3′UTR SNPs change miRNA–target interactions at the 3′UTR binding site, act as eQTLs for the targets in MS relevant tissues (e.g. PBMCs, lymphocytes) and have the lost/gained microRNAs expressed in the same MS relevant tissues. We are limited by study design and will not be doing the protein-level gain and loss experiments (condition 3). These criteria are highlighted in Additional file 2: Fig. S3.

We used the FiveX browser of eQTL catalogue [36] to identify the tissues in which our 3′UTR SNPs were acting as eQTLs for the predicted targets. We also checked our candidate SNPs within a more specific MS eQTL dataset which was provided alongside the MS GWAS [6] (Additional file 1: Table S11). We also identified the probability of those SNPs lie in regulatory regions within the genome through the probability score (best probability) and the type of regulatory site (RDB Rank) from RegulomeDB v.2.03 [74]. We also used the human.mirna.cellontology dataset from FANTOM5 [38] to check which cells our miRNAs were enriched/depleted in. In addition, checked the basal expression on the webtool Zenbu miRNA atlas (comparing microRNA expression across 0.5 low/10 medium/1000 highTPM) (Additional file 2: Fig. S3).

### microRNA gain/loss analysis

We used TargetScan 7.0 prediction algorithm locally to analyse microRNA-binding changes in variant vs reference 3′UTR sequences. The TargetScan miR_Family_Info.txt file was retained, while the reference and alternative 3′UTR sequences were formatted using SeqKit [66] to match and replace the 3′UTR file. We compared the predicted microRNA families compared between output files from the alternative and reference sequences in an R script.

## Supplementary Information

**Additional file 1: Table S1.** miRNA SNPs in summary statistics. **Table S2.** RNAfold rs1414273 effects. **Table S3.** MIR6891 validated targets lost. **Table S4.** MIR6891-predicted targets lost. **Table S5.** Candidate SNPs in flanking regions of miRNA precursors. **Table S6.** miRNA gain/loss rs6742. **Table S7.** Independent SNPs. **Table S8.** miRNA gain/loss independent SNPs. **Table S9.** Functional prioritisation. **Table S10.** miRNA-associated SNP candidates. **Table S11.** List of IMSGC data files referenced.

**Additional file 2.** Supplementary Figures 1–6.

## Data Availability

MS GWAS meta-analysis summary statistics are available at the IMSGC website: http://imsgc.net/. The data, which support the conclusions of this study, are included in this published article and its Additional files. Other datasets used and/or analysed during the current study are available from the corresponding author on reasonable request.

## Abbreviations

3′UTR: 3′ Untranslated region
CNS: Central nervous system
CSF: Cerebrospinal fluid
EAE: Experimental autoimmune encephalomyelitis
EBV: Epstein–Barr virus
GWAS: Genome-wide association studies
LD: Linkage disequilibrium
MHC: Major histocompatibility complex
MS: Multiple sclerosis
miRNA: MicroRNA
