# Supplementary material for: In silico prioritisation of microRNA-associated common variants in multiple sclerosis
Source: Hum Genomics. 2023 Mar 30;17:31. doi: 10.1186/s40246-023-00478-4 (PMC10061723; doi:10.1186/s40246-023-00478-4)
Supplement: Supplementary file 2 — Additional file 2. Supplementary Figures 1–6. [file 40246_2023_478_MOESM2_ESM.pdf]

# Supplementary Figure 1

**A**

| **Model** | **Reference ∆G** | **Alternative ∆G** | **Predicted Impact** |
|---|---|---|---|
| CEN | -71.9 | -71.3 | None |
| MEA | -81.5 | -82.7 | Flank |
| MFE | -85.2 | -84.6 | None |

**B**

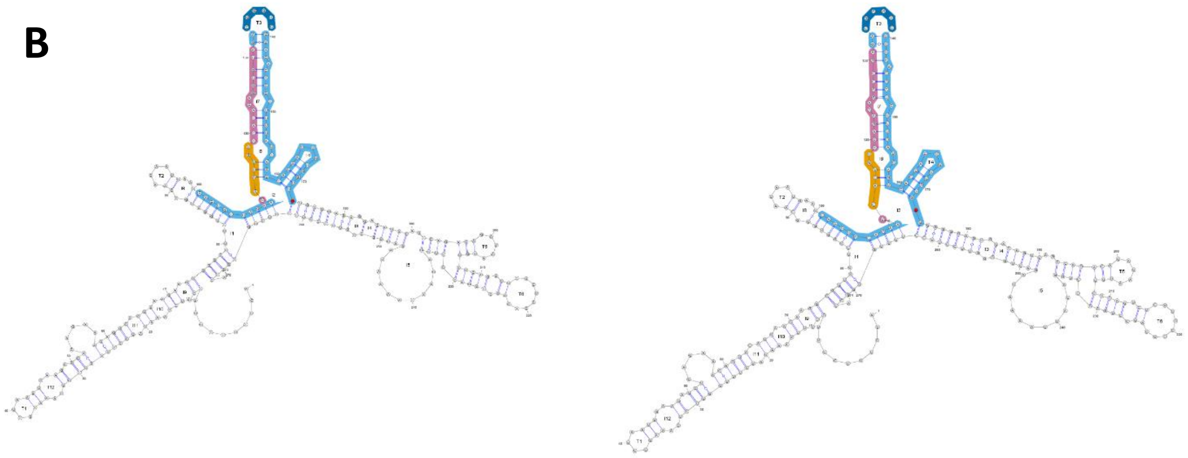

Reference Variant

**Supplementary Figure 1: A.** Table showing the predicted impact of rs2648841 on structural conformation of miR-1208. The MEA model predicts a change in the flank, while the other two predict no changes due to the SNP. **B.** Structural prediction by miRVaS showing the MFE folding of *MIR1208* alleles in rs2648841. There is no change to the overall structure of the microRNA

# Supplementary Figure 2

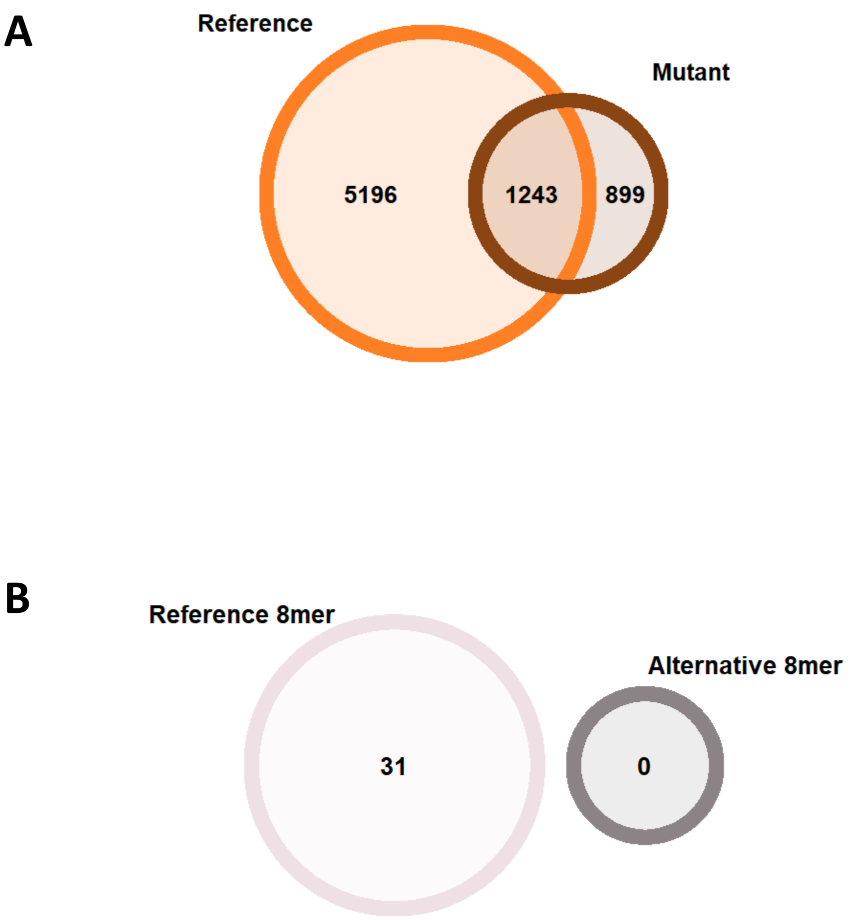


**Supplementary Figure 2:** Among the 3 candidate SNPs in miR-6891, we explored the target-binding consequences of the seed SNP rs2276448. We used TargetScan 7.0 algorithm to predict changes in 3'UTR target-binding ability between reference and variant seed sequences rs2276448 (CCUCAUC vs CCUCGUC in miR-6891-3p). In addition, we extracted validated targets of miR-6891 from miRTarBase (Huang et al., 2020). **A.** On comparing the pool of targets between the T and C alleles, we found over 1000 predicted and validated targets in common. However, the T allele binds nearly 6 times more targets than the C allele, suggesting that the T (risk) allele has more regulatory activity than the C allele. **B.** Similar trends are observed even after segregating the types of seed region interactions and validated targets from predicted targets. Even among canonical 8mer binding sites for validated targets, there is a distinct separation between the targets for each allele.

Those targets unique to the risk allele (ST3-4) have a broad range of biological functions. More importantly, the predicted targets which are not regulated by the C allele are significantly overrepresented in cellular components including postsynaptic structures, golgi membranes, neuron projection (Mi et al., 2017).

# Supplementary Figure 3

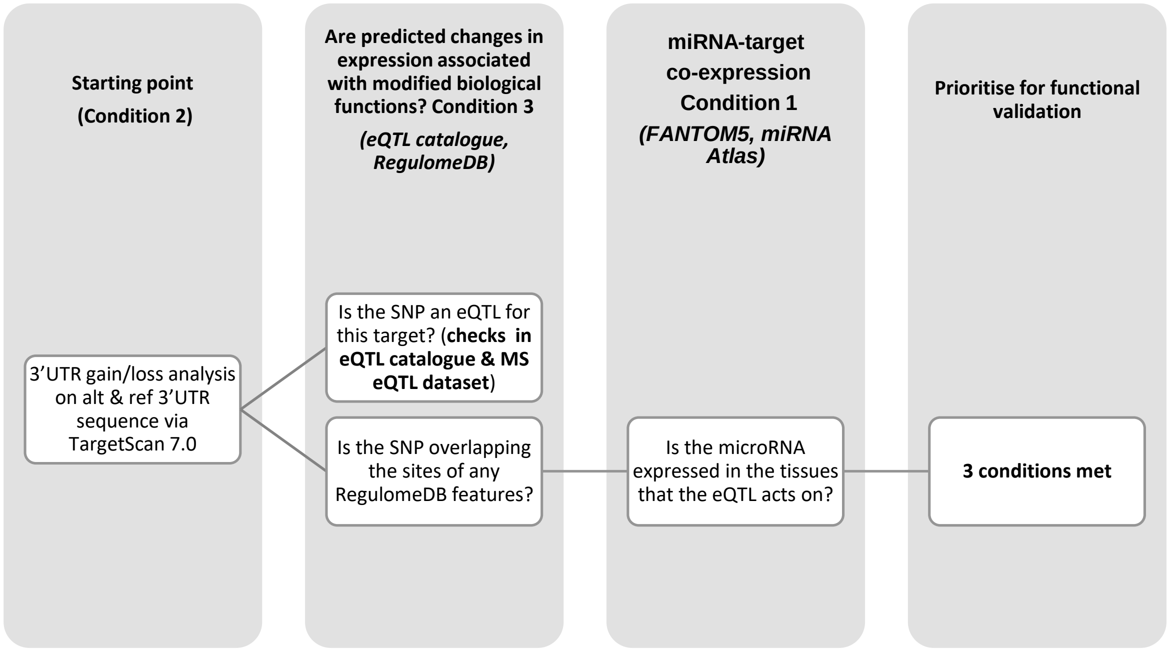


**Supplementary Figure 3:** microRNA-target validation pipeline. We aim to identify 3'UTR SNPs which meet 3 criteria. We adapted these criteria from microRNA-target validation guidelines (Kuhn et al., 2008; Elton and Yalowich, 2015; Riolo et al., 2021). **See methods (microRNA-target functional pipeline)**

Functionally relevant 3'UTR target SNPs were expected to meet these:

1) demonstration of microRNA & predicted target co-expression
2) direct interaction between miRNA and region on target
3) gain and loss experiments to show target protein interaction and
4) predicted changes have biological functions.

In short, we expected that functionally relevant 3'UTR SNPs would change miRNA-target interactions at the 3'UTR binding-site (via miRNA gain/loss analysis), act as eQTLs for the targets in MS relevant tissues (e.g PBMCs, lymphocytes), and have the lost/gained microRNAs expressed in the same MS relevant tissues. We are limited by study design and will not be doing the protein-level gain and loss experiments (condition 3).

# Supplementary Figure 4

A

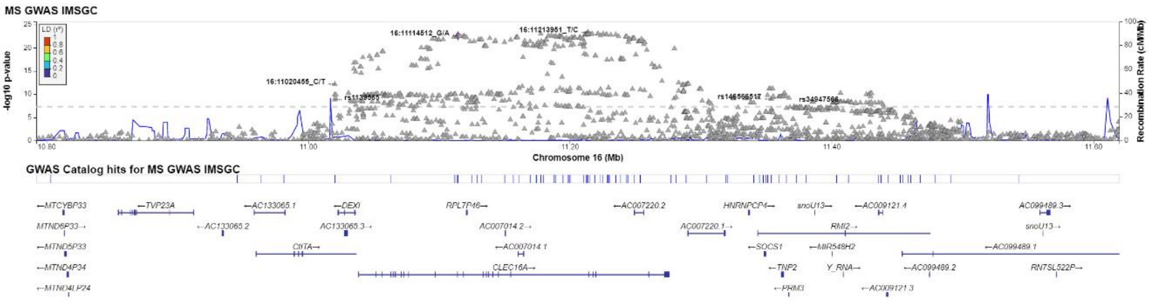


B

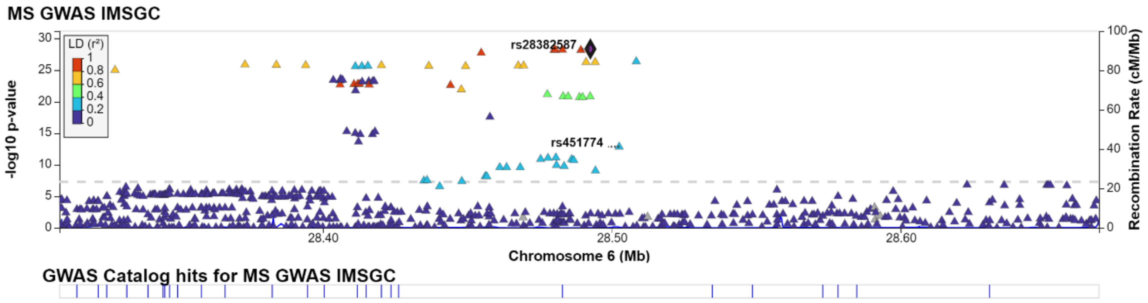


**Supplementary Figure 4:** Regional LocusZoom plots for the other independent SNPs which do not meet functional criteria. **A.** CIITA 3'UTR SNP rs11648656 (chr16:11020455) is among our independent SNPs. We show this in comparison to the IMSGC effect and discovery SNPs at this MS risk locus. The four (4) known MS SNPs rs2286974 (chr16:11114512), rs34947566, chr16:11353879 (rs146566517), chr16:11213951 tag in CLEC16A and RMI1. We did not prioritise rs11648656 as its significant eQTL activity has been shown in oesophageal and nerve tissue. **B**. Our candidate SNP rs451774 is independent. There are no other SNPs in this region that were prioritized among the genome-wide IMSGC SNPs. However, rs28382587 appears to be the SNP with the highest p-value in the region. Rs28382587 is intronic, within GPX6 but is not among the prioritised effects. The two SNPs are not in high LD (r2= 0.2643). We did not prioritise rs451774 further as its significant eQTL activity has been shown in adrenal glands, rather than directly on immune cell types and the changed microRNA is expressed in iPSCs (**ST9**). Rs17763689 *ATF7* is not represented in our LocusZoom plots.

# Supplementary Figure 5

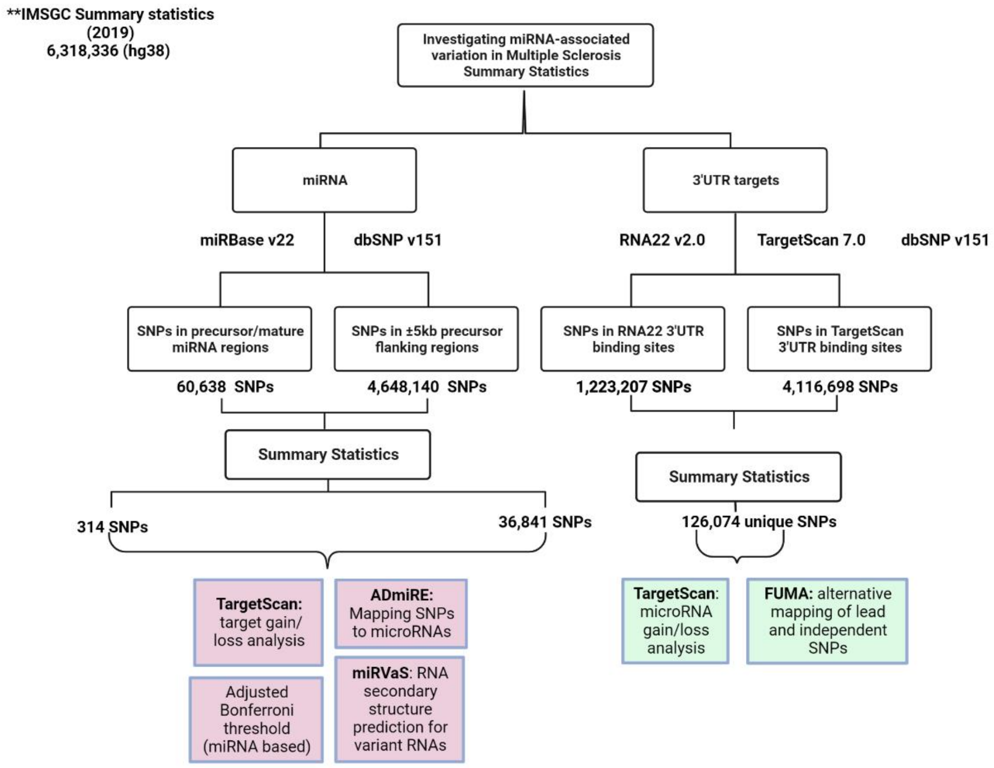


**Supplementary Figure 5:** Schematic diagram showing prioritisation of microRNA-associated variants using microRNA specific tools **(see Methods).**

# Supplementary Figure 6

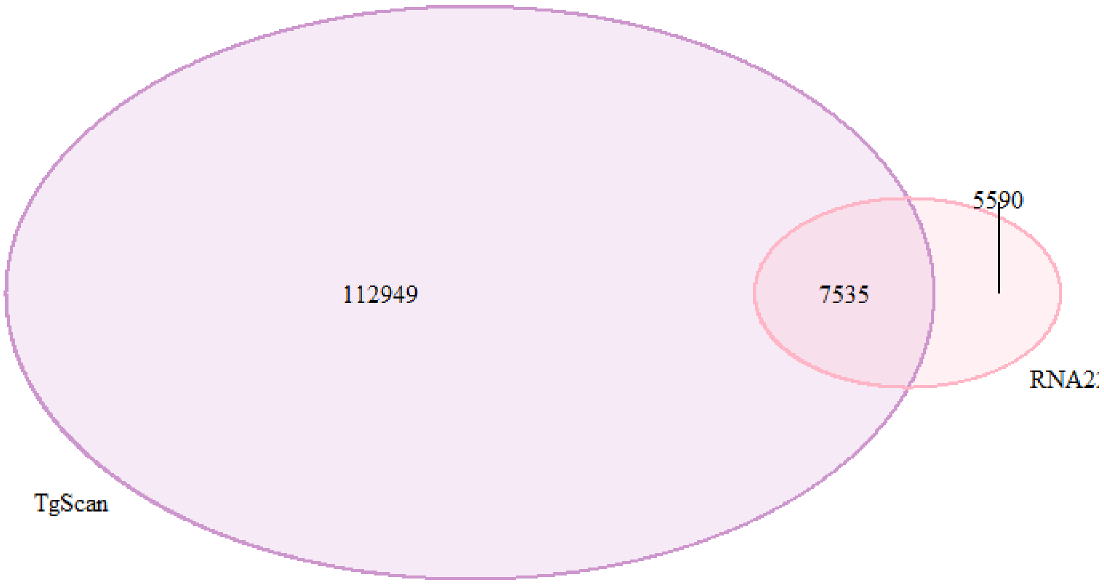


**Supplementary Figure 6:** Overlap between targets predicted by TargetScan and those with best probability from RNA22 **(see methods).** This intersection does not account for the different SNPs within the targets predicted.
